# Critical thinking development in undergraduate midwifery students: an Australian validation study using Rasch analysis

**DOI:** 10.1186/s12884-022-05303-9

**Published:** 2022-12-27

**Authors:** Amanda G. Carter, Amanda Müller, Michelle Gray, Dianne Bloxsome, Kristen Graham, Dolores Dooley, Linda Sweet

**Affiliations:** 1 School of Nursing and Midwifery, Faculty of Health, Griffith University, Griffith, Australia; 2 College of Nursing and Health Sciences, Flinders University, Adelaide, Australia; 3 School of Nursing and Midwifery, Edith Cowan University, Bunbury, Australia; 4 School of Nursing and Midwifery, Faculty of Health, Deakin University, Geelong, Australia; 5 Centre for Quality and Patient Safety, Western Health Partnership, Melbourne, Australia

**Keywords:** Critical thinking, Midwifery student, Rasch analysis, Critical thinking, Pre-registration, Evaluation

## Abstract

**Background:**

Well-developed critical thinking skills are required to provide midwifery care that is safe, evidence-based, and woman-centred. A valid, reliable tool to measure is required the application of critical thinking in midwifery practice. The Carter Assessment of Critical Thinking in Midwifery (CACTiM) has previously been psychometrically assessed using classical methods at a single site. This study aims to further evaluate the properties of CACTiM tools using Rasch analysis in a diverse group of midwifery students and preceptors.

**Methods:**

The CACTiM tools were completed by undergraduate midwifery students studying at three Australian universities and their preceptors. Midwifery students’ critical thinking was evaluated separately through student self-assessment and preceptor assessment and then matched. Rasch analysis was used to evaluate the validity of the tools.

**Results:**

Rasch analysis confirmed both the preceptor and student CACTiM tools demonstrated good reliability and unidimensionality. The items can differentiate between students’ ability to apply critical thinking in midwifery practice. Person reliability and item reliability were above .92 for both scales indicating excellent reliability and internal consistency. Several improvements were identified to the tools, including enhanced wording to some items, and reduction to a 5-point Likert scale. Through analysis of lower-scoring items, midwifery programs can identify curricula enhancements.

**Conclusion:**

The CACTiM student and preceptor tools are valid and reliable measures of critical thinking in midwifery practice. The tools can assess students’ critical thinking abilities and identify areas for development for individuals and across student cohorts through curricula enhancements.

**Supplementary Information:**

The online version contains supplementary material available at 10.1186/s12884-022-05303-9.

## Background

Midwifery decision-making is complex and therefore requires highly developed critical thinking skills. Critical thinking is a thoughtful process that is purposeful, disciplined, and self-directed to improve decisions and subsequent actions [1]. In making clinical decisions, midwives need to consider the best available evidence, contextualise the evidence to the individual woman, respect the woman’s preferences and needs, and sustain normal physiology where possible. This complexity is increased by the need to simultaneously provide care to both women and babies within a woman-centred framework, where the woman and midwife work in partnership and informed shared-decision making is facilitated, and pregnancy and birth are viewed as normal physiological processes [2]. Well-developed critical thinking skills are required to inform comprehensive professional judgement and effective problem-solving skills [3].

Midwifery students need to develop critical thinking skills and intellectual independence to inform decision-making [4]. However, there remains limited exploration surrounding these thinking processes [5–7] and their measurement within midwifery contexts [6].

A systematic review of the literature identified the need for discipline-specific tools measuring the application of critical thinking in midwifery practice [6]. In response to this deficit, the Carter Assessment of Critical Thinking in Midwifery (CACTiM) tools were developed (student and preceptor versions), which aimed to assess midwifery students’ critical thinking in midwifery practice. This involves two tools which use self-evaluation and focus on a student’s ratings of their own critical thinking [8] and the preceptor’s ratings of the student’s critical thinking [9]. The CACTiM student version has 25 items, and the preceptor version has 24 items measuring the distinct and complex aspects of critical thinking in midwifery practice. Both tools were developed using the staged model for tool development suggested by DeVellis [10], involving item generation, mapping draft items to critical thinking concepts, expert review to test content validity, pilot testing of the tool to a convenience sample of students and preceptors, and psychometric testing [8, 9]. Expert review of both tools resulted in a high content validity index of 0.97. Reliability was confirmed with a Cronbach’s alpha coefficient of 0.92 (student scale) and 0.97 (preceptor scale). Total and subscale scores correlated significantly for both tools. Sampling was from a single midwifery program at one university. Given these tools were tested with a homogonous sample, it was appropriate for larger and more diverse sample be used for further validation, using the more sophisticated testing of Rasch analysis.

Rasch analysis is both a model of measurement and a description of data. In this model the ideal features of data that defines successful measurement are featured, and the underpinning paradigm it is unlike other statistical models because the objective is to establish how well-observed data fits the model [11]. The Rasch model requires unidimensionality – a single quality measured along a single line of more or less of that quality. Rasch analysis allows the examination of the internal consistency of a tool, exposing the relationship between items and persons and enabling precise measurement. Rasch was used in this study to assess how consistently students of different abilities answer items related to critical thinking of varying difficulties. Rasch analysis also identifies if a tool is too easy or hard for a person or a group of respondents.

Rasch analysis provides information about the quality of measurement items and can rank items in order of difficulty [12]. This knowledge can be used to scaffold critical thinking activities so easier tasks are mastered before more complex tasks are attempted. Rasch analysis also analyses the level of difficulty of items. It identifies overlapping items that may measure the same concept or significant gaps where there are no question items providing a measurement of a particular concept in the continuum. This provides information for the tool creator to modify current items or create new items to address any redundancies or gaps [12].

The aim of this study was to evaluate further the properties of the CACTiM tools using Rasch analysis in a diverse group of midwifery students and preceptors.

## Methods

### Design and setting

The study was a cross-sectional online survey design. The research team was a collaboration of midwifery academics across Australia. Data was collected from three universities. Two of the universities offer a Bachelor of Midwifery program of three years, and the other university offers a four-year dual Midwifery and Nursing degree.

### Recruitment

A convenience sample of undergraduate midwifery students in their second, third, or fourth year from the three participating universities in Australia were invited to participate in this study. The cohort of students had completed at least 18 months of undergraduate midwifery study, including clinical and theoretical education at the time of participation. While students at one site were required to complete this survey as a part of their end-of-year clinical assessment, students in the other two research sites were invited to voluntarily participate in this study via student email and/or by a post in the online student portal. All potential participants were informed of the aims and procedures of the study, and a detailed participant information sheet was on the landing page of the online surveys. During the recruitment process, students were asked to seek the permission of their preceptor to provide their name and email address for recruitment purposes. The preceptor then received an auto-generated email about the study. Preceptors who agreed to participate completed the CACTiM preceptor version for the individual nominating student. For the purpose of this paper, the term preceptor will be used to describe the qualified midwife who supervises to the midwifery student during practice placement. It is recognised that a variety of terms are used in the literature to describe this role, including mentor, clinical facilitator, and clinical mentor.

### Data collection

Data were collected and managed using REDCap (Research Electronic Data Capture) [13], with each site managing their own data. Student and preceptor responses were downloaded independently and then matched with a study code to allow an exploration of rater severity and instrument dimensionality. All data were deidentified prior to analysis. Demographic details collected from student participants included age, year level, degree type, setting, and time on placement. Demographic information collected from preceptor participants included years of experience as a preceptor, qualifications, role, and time spent with the student. Note that preceptors may have supervised more than one student or may have declined participation. All participants were provided with instructions on how to complete the CATCiM tool and then provided with 25 (student) or 24 (preceptor) items to rank on a six-point Likert scale ranging between: strongly disagree, disagree, tend to disagree, tend to agree, agree, and strongly agree.

### Data analysis

The self-rated questionnaire data were collected from 270 midwifery students (first Excel spreadsheet) and preceptor-rated data for 197 students (second Excel spreadsheet). A third Excel dataset was created where the student and preceptor's answers to the 24 matching questions of the two versions of the tool were combined to explore rater severity and instrument dimensionality. Winsteps version 5.2.2.0 was used to analyse the data. This program was used to analyse unidimensionality, person, and item reliability and separation, map persons against item difficulty, item polarity, category measures, and item fit. These elements provided information about the reliability of the tool, how well each item fits within it, and if there were any structural anomalies in the tool.

### Ethical considerations

This study was approved by the ethics committees of each participating university and, where required, clinical sites where the preceptors were employed. The study was conducted in accordance with the ethical principles outlined in the Declaration of Helsinki and the Australian NHMRC guidelines. The study was carried out in accordance with the relevant regulations and guidelines. Participation was voluntary, and informed consent was gained when each participant (preceptor or student) completed the survey. The participant information sheet clearly stated consent was implied on completion of the survey. All identifying information was removed before being combined for analysis.

## Results

The sample consisted of 270 students and 197 preceptor responses from three universities. The available demographic data are shown in Table 1. Most students were enrolled in a Bachelor of Midwifery (81%) compared to a dual Bachelor of Nursing/Midwifery degree (19%) and were of an average age of 23 years. The majority of students were in the 3^rd^ year of their degree (63%). Of preceptors, most held a Bachelor’s degree (67%), with nearly one-third having postgraduate qualifications (31%). On average, preceptors had 39 h of contact with a student prior to completing the CACTiM tool and an average of nine years experience in the preceptor role.

**Table 1:** Demographic data of participants

Variable		Student	
	Category	Count	Column N %
	Total	270	100%
Student degree	Bachelor of Midwifery (3 year degree)	126	81%
	Bachelor of Nursing/Midwifery (4 year degree)	30	19%
Student year	Second Year	56	36%
	Third Year	98	63%
	Fourth Year	2	1%
Setting	Domiciliary/community/home visit	3	1%
	Midwife group practice	33	16%
	Postnatal ward	34	17%
	Birth suite	124	61%
	Antenatal clinic/ward	7	3%
	Birth Centre	1	0%
Variable		Preceptor	
	Category	Count	Column N %
	Total	197	100%
Preceptor role	Clinical facilitator	7	4%
	Midwifery educator	14	7%
	Preceptor/mentor	175	89%
Qualifications	Hospital	4	2%
	Bachelor	132	67%
	Postgraduate study	60	31%

### Rasch analysis

The majority of survey items were answered, with only two incidents of a missing answer to a question. The results for the Rasch analysis section will be arranged with student and then preceptor data will be presented sequentially under each sub-section. The items for both versions of the CACTiM (Student and Preceptor) tools are included in Table 2.

**Table 2:** Ranking of items as most difficult to easiest to endorse by students and preceptors where 1 = most difficult to 25 = easiest

Item No	CACTiM Student items	CACTiM Preceptor items	Difficulty ranking student	Difficulty ranking preceptor
1	I explore the woman's preferences of care and plan care accordingly	Explores the woman's preferences of care and plan care accordingly	21	20
2	I sequence care and education to meet the individual needs of the woman	Sequences care and education to meet the individual needs of the woman	17	21
3	I choose relevant literature and education strategies to facilitate the woman's decision making	Suggests relevant literature and education strategies to facilitate the woman's decision making	8	1
4	I share relevant evidence and clinical guidelines related to the woman’s individual circumstances	Shares relevant evidence and clinical guidelines related to the woman’s individual circumstances	9	10
5	I use evidence to plan care according to the woman’s individual circumstances	Uses evidence to plan care according to the woman’s individual circumstances	14	7
6	I often instinctively know what type of care is right for the woman	Demonstrates insight in providing individualised care to the woman	2	24
7	I liaise and negotiate with colleagues at different levels about processes to optimise outcomes for the woman	Liaises and negotiate with colleagues at different levels about processes to optimise outcomes for the woman	10	3
8	I consult resources (e.g. literature, guidelines, etc.) to improve care for the woman	Consults and utilises resources (e.g. literature, guidelines, etc.) to improve care for the woman	12	11
9	If problems arise when caring for the woman I try to seek the root cause	Seeks the root cause if problems arise whilst caring for the woman	13	9
10	I explore multiple solutions to a given situation	Effectively explores multiple solutions to a given situation	7	13
11	I seek clarification about clinical procedures or practice that appears inappropriate or unnecessary	Seeks clarification about interventions that appear inappropriate or unnecessary	15	19
12	Where needed, I negotiate a collaborative intervention plan with relevant health care providers	Where needed, negotiates a collaborative intervention plan with relevant health care providers	6	4
13	I can provide the rationale for following (or departing from) established guidelines and policies	Demonstrates an understanding of the rationale for following (or departing from) established guidelines and policies	5	17
14	I can recognise non-evidence based or non-woman centred practice by self and others	Recognises non-evidence based or non-woman centred practice by self and others	16	6
15	I voice my concerns about non-evidence based or non-woman centred practices by self and others	Voices concerns about non-evidence based or non-woman centred practices by self and others	3	8
16	I identify organisational/service improvement opportunities	Identifies organisational/service improvement opportunities	1	5
17	I question the ‘unwritten rules’ in midwifery practice that are not evidence-based	Questions the ‘unwritten rules’ in midwifery practice that are not evidence-based	4	2
18	I continually analyse my own strengths and limitations in skills, knowledge and experience	Analyses own strengths and limitations in skills, knowledge and experience	24	16
19	I address my limitations in skills, knowledge and experience	Addresses own limitations in skills, knowledge and experience	19	18
20	I initiate professional dialogue around midwifery practice	Initiates professional dialogue around midwifery practice	11	14
21	I evaluate my practice and its effect on the woman and others	Evaluates own practice and its effect on the woman and others	18	15
22	I adjust my practice based on feedback from the woman and others	Adjusts own practice based on feedback from the woman and others	25	22
23	I recognise my attitudes, biases and values and their potential impact on practice	Recognises own attitudes, biases and values and their potential impact on practice	20	12
24	I debrief with a professional colleague following complex situations to improve my future practice	Debriefs with a professional colleague following complex situations to improve my future practice	23	23
25	I apply knowledge from past experiences to present situations	-	22	

### Dimensionality

The eigenvalue units of the standardised residual variance provide information about whether a tool measures one dimension or concept, and ideally, the variance should be above 20%. As a guideline, the unexplained variance in contrasts 1–5 should be less than 3.0 [14]. The eigenvalue for students was 57.24, with a variance of 56.3% and an unexplained variance in the first contrast of 2.56. The eigenvalue for preceptors was 55.14, with an explained variance of 56.5% and an unexplained variance in the first contrast of 3.45. These values indicate the tools can be treated as unidimensional instruments. The eigenvalue tables are available in supplementary data 1.

### Person and item reliability

Person and item reliability determines what proportion of the variance found among items or persons is not due to error (randomness), and thus the results could be replicated where the same people would score the same way on another set of related items on the same dimension [12]. The values range from 0–1, with 1 being the most desirable. Among students and preceptors, the person reliability was 0.92 and 0.93, respectively. Similarly, item reliability was 0.99 and 0.92, respectively. The figures can be interpreted similarly to Cronbach’s alpha and indicate the instruments have excellent internal consistency. Among students and preceptors, person separation was 3.47 and 3.73, respectively, and item separation was 9.41 and 3.33, respectively. This means both the persons and items are functioning to differentiate between people and abilities of different levels, well above the acceptable standard of around 0.8 [15]. An inspection of the point-measure correlations for both groups shows all positive relationships between the range of 0.54 to 0.78, indicating the responses align with students’ critical thinking abilities (see supplementary data 2).

### Wright maps

**Fig. 1:**
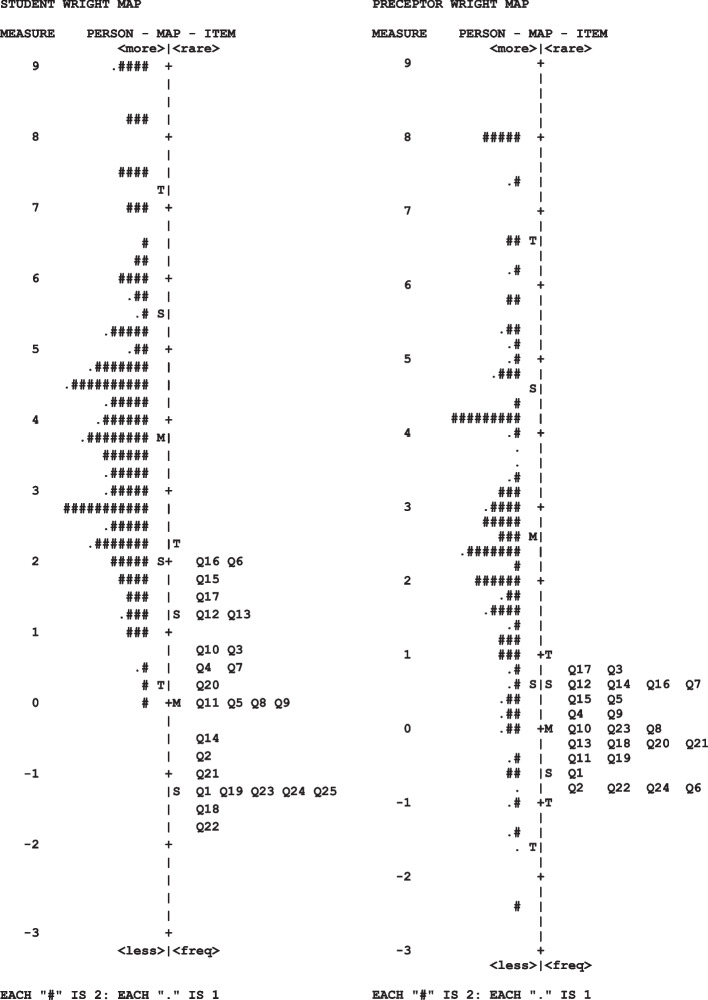
Wright Maps for student and preceptor responses

A Wright Map is a visual representation of the continuum being measured [12]. In the Wright Maps shown in Fig. 1, the lower performing persons are indicated at the bottom of the left-hand side and more frequent high-scoring questions (easier to endorse) at the lower right of both maps. The distribution of higher-performing students is at the top left, and the harder questions (harder to endorse) are near the top right of both maps.

The Wright maps show how item difficulty is ranked along the continuum of critical thinking, from more difficult to endorse at the top (such as question 16) to easier to endorse at the bottom (such as question 22). The item difficulty sits closer together for preceptors in a small range of -1 and + 1 logits, and for students, the item difficulty occurs in a larger range between -2 to + 2 logits. This means the item difficulty is less easily distinguished for preceptors than students. The clustering of questions horizontally indicates redundancies because several items are essentially asking about the same level of critical thinking (e.g., questions 1, 19, 23, 24, and 25 among the students). In terms of ability, students rate themselves as having higher-ability, and preceptors rate student ability along with a wider range of logit scores. The “M” (mean) shows the typical student has a mean person measure of about 3.7 logits, whereas preceptors tend to rate student ability about 2.7 logits, or a logit lower. Note that in both groups, the person mean sits well above the second standard deviation of items (outside the confidence interval).

### Item difficulty and rating scale performance

The ranking of difficulty for items in both tools is provided in Table 2. The item-category measures are available in supplementary data 3. The counts are given below in Tables 3 and 4, which show a heavy skew towards answering ‘agree’ (4) or ‘strongly agree’ (5) to all questions by students and preceptors, with some Likert categories being almost redundant, such as ‘strongly disagree’ (0) and ‘disagree’ (1), where there were very few responses. The Likert option of ‘tend to agree’ was used more by students than preceptors.

**Table 3:** Students—summary of category structure

CATEGORY		OBSERVED		OBSVD	SAMPLE	INFIT	OUTFIT	ANDRICH	CATEGORY	
LABEL	SCORE	COUNT	%	AVRGE	EXPECT	MNSQ	MNSQ	THRESHOLD	MEASURE	
0	0	2	0	2.23	-1.09	3.18	8.65	NONE	( -5.07)	0
1	1	45	1	-.19^a^	-.47	1.24	1.43	-3.91	-2.73	1
2	2	158	2	.37	.46	.94	.92	-1.28	-1.09	2
3	3	1103	16	1.71	1.73	.98	.98	-.87	.49	3
4	4	2752	41	3.34	3.35	.97	.92	1.60	3.05	4
5	5	2688	40	5.52	5.51	1.01	1.01	4.47	(-5.61)	5

^a^disordered

**Table 4:** Preceptors—summary of category structure

CATEGORY		OBSERVED		OBSVD	SAMPLE	INFIT	OUTFIT	ANDRICH	CATEGORY	
LABEL	SCORE	COUNT	%	AVRGE	EXPECT	MNSQ	MNSQ	THRESHOLD	MEASURE	
0	0	3	2	2.51	-1.79	5.74	9.90	NONE	( -2.62)	0
1	1	1	1	-2.38^a^	-1.37	.14	.13	.03	-1.92	1
2	2	1	1	-1.51	-.83	.00	.00	-.59	-1.49	2
3	3	11	6	-.58	.11	1.33	.78	-2.30	-1.02	3
4	4	92	47	1.78	1.81	.88	.79	-.70	.94	4
5	5	89	45	4.04	4.05	1.10	1.04	3.55	(-4.14)	5

^a^disordered

Tables 3 and 4 provide a range of information. For the students, the sample expectation column values that represent the Rasch model expected values are significantly different to the observed averages, indicating it was hard to distinguish between answer options (strongly disagree to strongly agree). The Andrich threshold columns show the points where the probability of choosing another category is higher than choosing the previous category. The distance between two thresholds should be at least 1.2 logits, but the Andrich thresholds between, for example, 2 and 3 are below this, so the thresholds are small (for example, students did not discriminate well between ‘tend to disagree’ and ‘tend to agree’). These results indicate that the Likert options could be examined and condensed.

### Item infit and outfit

The mean square infit and mean square outfit data show how well items operate with other items in measuring a dimension. The infit and outfit statistical analysis fell within acceptable ranges of 0.75 to 1.3) [12], with a few values in minor excess of this (see supplementary data 4), indicating the items worked suitably well with other items to measure critical thinking.

### Comparing the groups: differential item functioning

The student and preceptor versions of the survey tool are closely matched in wording and content, and therefore a speculative analysis of group differences was conducted. An examination of the combined model revealed excellent person and item separation, positive point-measure correlations, similar fit statistics to the single models, and the Andrich thresholds improved over the single group models. These are all excellent indices that analysis between students and preceptors is viable.

**Fig. 2:**
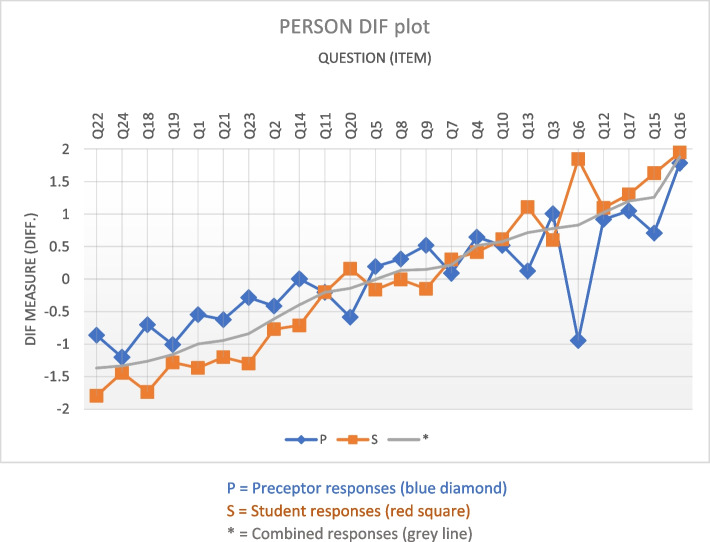
Differences in measurements by group. The more difficult-to-endorse questions have higher DIF points of measurement, and the more easy-to-endorse questions have lower DIF points of measurement. Thus preceptors found it harder to rate more question items harder than students, with some notable exceptions where they crossover

The main question of the combined model was whether there were differences coming from persons and if the mean measures between groups were the same. Figure 2 demonstrates the agreement and differences between student and preceptor ratings on each question, and Table 5 shows the differences by question. This is interpreted similarly to the Wright Map, so the higher the line goes, the more difficult each item is to endorse, and the easier-to-endorse questions are toward the bottom. Thus, for question 6, a notably different question from the others, the preceptors found it very easy to endorse student performance, yet students found it very difficult to endorse their own performance as good on that question.

**Table 5:**
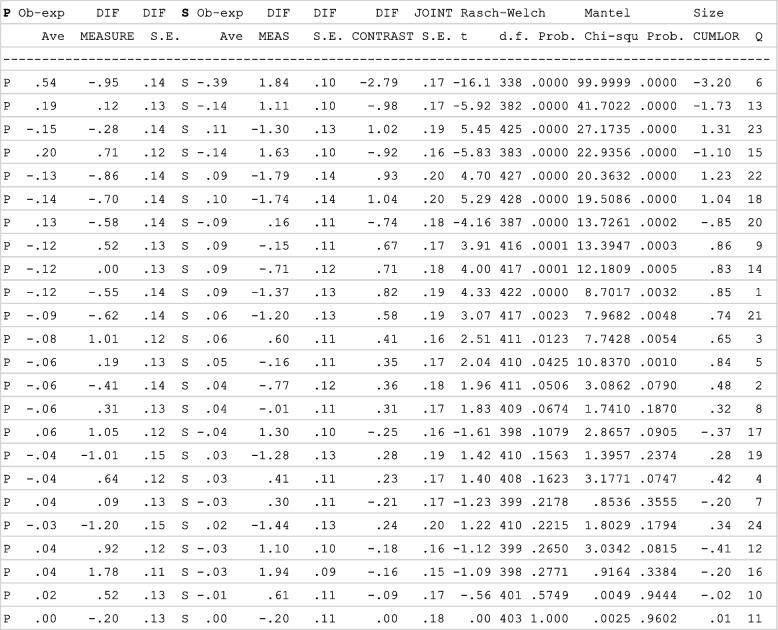
Group differences for each item

When t-tests were run on the differences between groups, significant differences (Rasch-Welsch probability column) were found for 13 question items. The ‘DIF contrast’ tells us the log-odds unit difference between groups (identifying the most controversial items), where positive values mean the item is harder for preceptors to endorse students on this item. For example, in item 1 ‘DIF measure’ for preceptors (-0.55) and students (-1.37), the contrast is 0.82, showing it was harder for preceptors than students to endorse.

## Discussion

Rasch analysis revealed good reliability and unidimensionality for both the student and preceptor CACTiM tools. The items can differentiate between student’s ability to apply critical thinking in midwifery practice. Person reliability and item reliability were above 0.92 for both scales indicating excellent reliability and internal consistency. The analysis also confirmed the more difficult items (lower scoring) aligned with those found in the initial validation studies [8, 9]. During initial tool development, items were mapped to the core concepts of critical thinking to ensure all components of critical thinking were represented [9]. The lower-scoring items in this study were originally mapped to higher-order concepts of critical thinking of transforming knowledge (recognising theory from practice, testing theory in practice, and synthesis) and discriminating (classifying, choosing relevance/irrelevance, recognising gaps/inconsistencies, prioritising) [16]. This further confirmed these items represent more discerning factors in the application of critical thinking.

The CACTiM tools have the capacity to identify individual and group strengths and weaknesses, which may then be addressed in curricula. An identified difficult item from this study was item 16, which relates to recognising an organisation/service improvement. This item aims to measure students’ capability to question and identify practice improvements that will benefit the organisation and/or improve outcomes for women and babies [9]. This skill requires higher-order critical thinking skills and entails being able to speak up and advocate for change in an environment where conformity is encouraged to promote student enculturation [17]. With the recognition that the transformation of maternity services is long overdue, the ability to recognise the need for change and implement improved practice is essential. Through the administration of the CACTiM tools, individual midwifery programs can identify difficult items for students and use these to drive curricula change and improvement. For example, one of the universities involved in this study has already responded to the difficulty represented by item 16 and recently implemented an assessment item where final-year students identify and develop an implementation plan for practice improvement that promotes optimal birth practices. Two further difficult items identified (items 17 and 12) may indicate greater focus is needed in curricula on developing students’ ability to question professional practice, negotiate, and collaborate with other practitioners. Items 17 and 12 are related to the identification and questioning of ‘unwritten rules’ in midwifery practice and negotiation and collaboration to develop an intervention plan. Identification and questioning of ‘unwritten rules’ are vital critical thinking components as ‘unwritten rules’ are often ingrained practices that hinder evidence-based care, with care being based on tradition rather than current best evidence, contributing to less-than-optimal care for women and babies [9]. Questioning these ‘unwritten rules’, and collaboration and negotiation of care are complex skills to develop and require exceptional communication skills, cooperation, consultation, appropriate referral, and shared decision-making to ensure safe care is provided [18].

Conversely, easier items may indicate areas of the curricula that are either taught well or easier to master. Items related to reflection tended to score highly from both students and preceptors – an example is item 22: ‘I adjust my practice based on feedback from the woman and others’. This may indicate that the current learning and teaching strategies related to reflection are highly effective. The three universities involved in this study all teach reflective practice throughout the program as a mechanism to continually improve practice. Providing the students with a structured model of reflection, such as the Bass Model of Holistic Reflection, seems to be highly effective in teaching reflective practice in midwifery students [19].

Analysis using the Wright map identified some clustering of items which may indicate that several items are measuring the same levels within critical thinking. This clustering was identified within questions 1, 19, 23, 24 and 25 on the student survey. On further examination, these items are theoretically and conceptually unique, and although they may measure the same levels of critical thinking, they measure distinct concepts. Removal of one or more of these items may result in important concepts of critical thinking being underrepresented in the CACTiM tools, therefore all items were retained.

Comparisons of student and preceptor ratings determined that students often rated themselves higher in relation to their critical thinking abilities than their preceptor. Student self-assessment is a recognised pedagogical strategy to promote self-regulated and self-determined learning [20]. However, the accuracy of student self-assessment is questioned, and becomes more inaccurate when students’ self-assessment contributes toward their final marks/grades [21]. Although the midwifery students in this study were not given a final ‘score’ for the CACTiM assessment where use of the tool was mandatory, the assessment result contributed to the end-of-semester clinical assessment discussion, identifying the students’ potential areas of strength and areas for further development. This may have resulted in an overinflation of students’ self-assessment for this cohort of students. However, the ability to self-assess practice and competence is an essential skill for midwifery students as they prepare to become accountable midwifery practitioners [22]. The design of the CACTiM process whereby the preceptor also provides feedback on the application of critical thinking in practice adds to the validity of the assessment. The two tools offer a multi-method approach which provides an opportunity for the student and preceptor (and, at times Clinical Facilitator/Practice Lecturer) to discuss any discrepancies and identify possible areas for development.

The Rasch analysis showed that both students and preceptors scored the students well on most items. Given that the entry requirements into midwifery programs in Australian universities are usually high (e.g., an ATAR above 90), midwifery students are likely to be high academic performers. In addition, most of the students that completed the tool (64%) were in the third or fourth year of their degree and would be expected to demonstrate critical thinking in practice ready to graduate. Using Rasch analysis has provided valuable information regarding item difficulty, which could be used to scaffold the tool in the future and assign different weightings to individual items according to the degree of difficulty. Currently, students are not allocated a final score when using the tool. Instead, areas for improvement are identified and discussed. However, a score could be potentially assigned with expected levels of achievement according to each year level. For example, easier items could be allocated a higher weighting earlier in the degree and then a lower weighting in the final year and more difficult items could be assigned a lower weighting in early years and a higher weighting in the final year.

Analysis of the item Likert categories identified little discrimination between the two options of ‘tend to agree’, ‘tend to disagree’. Therefore, it is recommended in future iterations of the tool the current 6-point Likert scale is decreased to a 5-point Likert Scale of ‘strongly disagree’,’disagree’, ‘neither agree or disagree’, ‘agree’, and ‘strongly agree’. Providing a midpoint on a Likert scale allows respondents to choose a response in the mid-range when neither of the other options are appropriate [10]. Whilst it was identified that the strongly disagree option was not used, given the impact of deficits in critical thinking and consequent poor decision-making, it is important to maintain this option to identify and flag poor or unsafe practice.

Significant differences were found between student and preceptor responses for some items. Adjusting the wording of these items may improve comprehension by respondents and improve the differential item function. For example, one of the items with this discrepancy was item 6, where the student item is worded ‘I often instinctively know what type of care is right for the woman’ and the preceptor item is worded ‘Demonstrates insight in providing individualised care to the woman’. This student item was originally designed to measure the critical thinking concept of intuition [8]. Intuition is a complex process and, when used in decision-making, is often considered in the realm of an ‘expert’ practitioner who no longer uses formal analytical professional judgement processes [8]. A recently published international consensus definition of critical thinking in midwifery practice recognised intuition as a core aspect of critical thinking in midwifery and defined intuition as a ‘sense of knowing without the conscious use of reason, relying on exquisite sensitivity to pattern recognition and heuristics based on prior experience’ [2]. The matching preceptor statement for item six uses the term ‘insight’ which may be interpreted very differently than ‘instinctively’. The definition of ‘insight’ according to the Britannica dictionary is ‘the ability to understand people and situations in a very clear way’[23] p. 1. Therefore, this implies a lower level of cognitive thought than intuition and this item may be interpreted is simply referring to an understanding regarding care provided. It is recommended the wording of preceptor item 6 is altered to improve clarity to ‘Often appears to instinctively know what type of care is right for the woman’.

Item 13 also had significant differences between student and preceptor responses. This item, too may be improved with a simple wording change to the preceptor item. The student item reads, ‘I can provide the rationale for following (or departing from) established guidelines and policies’. In contrast, the preceptor item reads, ‘Demonstrates an understanding of the rationale for following (or departing from) established guidelines and policies’. A change in the preceptor item wording to improve congruency to the student item is recommended to ‘Can provide the rationale for following (or departing from) established guidelines and policies’.

### Strengths and limitations

This is the first published Rasch analysis of tools designed to measure the application of critical thinking in midwifery practice. Although the sample size is acceptable, a more diverse and larger sample size may have further enhanced the analysis. The original plan for this study was the involvement of five universities across Australia and New Zealand. Due to workload issues and challenges with student recruitment as a direct result of COVID-19, only data from three Australian universities could be included. This may affect the generalisability of this study as culture may impact teaching approaches used and critical thinking development. It is recommended that this study be undertaken with a larger, more culturally diverse sample. Although all students from one university completed the CACTiM tool as part of their clinical assessment, it is possible students from the other two universities may have been less confident or had different expectations on their time, impacting their decision to participate. It is recommended for future studies that the CACTiM tools are embedded into the curriculum prior to a study to ensure students are more familiar with the tools.

## Conclusion

This study has further demonstrated the reliability and validity of the CACTiM student and preceptor tools. Using the sophisticated psychometric method of Rasch analysis has identified recommended improvements to the tools to enhance their use in the future. These improvements include reducing the 6-point Likert scale to a 5-point scale and wording alterations of items to improve the differential item function and enhance the congruence of student and preceptor items. The CACTiM tools not only provide a reliable tool to measure the application of critical thinking in midwifery practice, analysis of a student cohort can also identify possible curricula enhancements. Using Rasch analysis, difficult items have been identified, and specific learning, teaching, and assessment strategies can be implemented to enhance midwifery students’ critical thinking skills in these areas.

## Supplementary Information

**Additional file 1:** **Supplementary data 1.** Eigenvalues. **Supplementary data 2.** Correlation Order Tables. **Supplementary data 3.** Empirical Item-Category Measures. **Supplementary data 4.** item misfit order.

## Data Availability

The analytic files are provided in the supplementary file. The datasets generated and/or analysed during the current study are not publicly available as we do not have ethics approval to share the raw data but are available from the corresponding author on reasonable request.
