# Supplementary material for: Critical thinking development in undergraduate midwifery students: an Australian validation study using Rasch analysis
Source: BMC Pregnancy Childbirth. 2022 Dec 27;22:972. doi: 10.1186/s12884-022-05303-9 (PMC9793377; doi:10.1186/s12884-022-05303-9)
Supplement: Supplementary file 1 — Additional file 1: Supplementary data 1. Eigenvalues. Supplementary data 2. Correlation Order Tables. Supplementary data 3. Empirical Item-Category Measures. Supplementary data 4. item misfit order. [file 12884_2022_5303_MOESM1_ESM.docx]

### Supplementary data 1: Eigenvalues

Table of student standardized residual variance in Eigenvalue units = ITEM information units

Eigenvalue Observed Expected

Total raw variance in observations = 57.2415 100.0% 100.0%

Raw variance explained by measures = 32.2415 56.3% 56.5%

Raw variance explained by persons = 17.0242 29.7% 29.8%

Raw Variance explained by items = 15.2172 26.6% 26.7%

Raw unexplained variance (total) = 25.0000 43.7% 100.0% 43.5%

Unexplned variance in 1st contrast = 2.5552 4.5% 10.2%

Unexplned variance in 2nd contrast = 2.1764 3.8% 8.7%

Unexplned variance in 3rd contrast = 1.7651 3.1% 7.1%

Unexplned variance in 4th contrast = 1.4103 2.5% 5.6%

Unexplned variance in 5th contrast = 1.3600 2.4% 5.4%

Table of preceptor standardized residual variance in Eigenvalue units = ITEM information units

Eigenvalue Observed Expected

Total raw variance in observations = 55.1383 100.0% 100.0%

Raw variance explained by measures = 31.1383 56.5% 57.2%

Raw variance explained by persons = 21.1425 38.3% 38.8%

Raw Variance explained by items = 9.9957 18.1% 18.4%

Raw unexplained variance (total) = 24.0000 43.5% 100.0% 42.8%

Unexplned variance in 1st contrast = 3.4521 6.3% 14.4%

Unexplned variance in 2nd contrast = 2.5743 4.7% 10.7%

Unexplned variance in 3rd contrast = 1.8249 3.3% 7.6%

Unexplned variance in 4th contrast = 1.5248 2.8% 6.4%

Unexplned variance in 5th contrast = 1.3743 2.5% 5.7%

### Supplementary data 2: Correlation Order Tables

**Student Item Statistics – Correlation Order**

----------------------------------------------------------------------------------

PERSON: REAL SEP.: 3.47 REL.: .92 ... ITEM: REAL SEP.: 9.41 REL.: .99

-------------------------------------------------------------------------------------------

|ENTRY TOTAL TOTAL JMLE MODEL| INFIT | OUTFIT |PTMEASUR-AL|EXACT MATCH| |

|NUMBER SCORE COUNT MEASURE S.E. |MNSQ ZSTD|MNSQ ZSTD|CORR. EXP.| OBS% EXP%| ITEM |

|------------------------------------+----------+----------+-----------+-----------+------|

| 18 1242 270 -1.61 .14|1.04 .47|1.03 .21| .52 .55| 73.9 73.2| Q18 |

| 24 1222 269 -1.32 .13|1.28 2.72|1.16 .84| .52 .57| 73.5 71.6| Q24 |

| 23 1217 270 -1.18 .13|1.05 .51| .99 -.01| .54 .58| 71.6 70.7| Q23 |

| 25 1223 270 -1.28 .13|1.09 .92|1.09 .50| .54 .57| 70.9 71.3| Q25 |

| 22 1245 270 -1.67 .14| .97 -.29| .83 -.71| .55 .54| 76.2 73.5| Q22 |

| 1 1221 270 -1.25 .13|1.07 .73|1.25 1.30| .58 .57| 76.6 71.0| Q1 |

| 19 1216 270 -1.17 .13|1.06 .61| .88 -.60| .58 .58| 69.0 70.6| Q19 |

| 14 1179 270 -.60 .12|1.09 .98|1.14 1.01| .58 .62| 65.9 67.2| Q14 |

| 11 1142 270 -.10 .11|1.21 2.20|1.13 1.07| .62 .65| 61.7 64.6| Q11 |

| 21 1211 270 -1.09 .13| .79 -2.42| .93 -.36| .63 .59| 76.6 70.0| Q21 |

| 6 956 270 1.92 .10|1.38 3.70|1.50 4.75| .64 .74| 47.5 57.8| Q6 |

| 7 1098 269 .39 .11|1.38 3.64|1.32 2.78| .64 .68| 56.5 63.2| Q7 |

| 2 1183 270 -.66 .12| .71 -3.51| .63 -2.89| .69 .62| 73.9 67.6| Q2 |

| 8 1127 270 .09 .11| .94 -.63| .93 -.63| .69 .66| 64.4 63.8| Q8 |

| 9 1138 270 -.05 .11| .89 -1.16| .91 -.73| .69 .65| 68.6 64.3| Q9 |

| 20 1114 270 .25 .11| .87 -1.45| .93 -.62| .70 .67| 70.1 63.4| Q20 |

| 17 1012 270 1.38 .10|1.14 1.42|1.09 .95| .70 .72| 58.2 60.2| Q17 |

| 15 979 270 1.71 .10|1.03 .38|1.05 .52| .71 .73| 57.1 58.7| Q15 |

| 16 945 270 2.02 .10|1.02 .26|1.07 .74| .72 .74| 54.8 57.5| Q16 |

| 10 1076 270 .70 .11| .82 -2.01| .79 -2.18| .72 .69| 64.8 62.1| Q10 |

| 5 1139 270 -.06 .11| .83 -1.98| .76 -2.15| .72 .65| 71.3 64.3| Q5 |

| 4 1093 270 .50 .11| .87 -1.37| .84 -1.54| .74 .68| 65.5 62.7| Q4 |

| 12 1032 270 1.18 .10| .97 -.29| .94 -.66| .74 .71| 62.8 60.7| Q12 |

| 3 1077 270 .69 .11| .76 -2.74| .82 -1.83| .74 .69| 68.6 62.1| Q3 |

| 13 1031 270 1.19 .10| .77 -2.57| .74 -2.93| .76 .71| 67.8 60.7| Q13 |

|------------------------------------+----------+----------+-----------+-----------+------|

| MEAN 1124.7 269.9 .00 .11|1.00 -.07| .99 -.13| | 66.7 65.3| |

| P.SD 90.9 .3 1.13 .01| .18 1.90| .19 1.69| | 7.4 4.9| |

-------------------------------------------------------------------------------------------

**Preceptor Item Statistics – Correlation Order**

------------------------------------------------------------------------------------

PERSON: REAL SEP.: 3.73 REL.: .93 ... ITEM: REAL SEP.: 3.33 REL.: .92

----------------------------------------------------------------------------------------------

|ENTRY TOTAL TOTAL JMLE MODEL| INFIT | OUTFIT |PTMEASUR-AL|EXACT MATCH| |

|NUMBER SCORE COUNT MEASURE S.E. |MNSQ ZSTD|MNSQ ZSTD|CORR. EXP.| OBS% EXP%| ITEM G |

|------------------------------------+----------+----------+-----------+-----------+---------|

| 1 849 197 -.52 .13|1.80 3.81|2.05 4.71| .54 .64| 73.3 70.5| Q1 0 |

| 20 851 197 -.15 .15|1.14 1.30|1.14 .91| .65 .69| 67.4 70.5| Q20 0 |

| 18 857 197 -.20 .14|1.14 1.32|1.10 .60| .65 .68| 66.3 68.1| Q18 0 |

| 7 814 197 .66 .14|1.21 1.91|1.36 2.56| .67 .72| 59.4 66.9| Q7 0 |

| 24 881 197 -.79 .16| .95 -.46| .84 -.78| .67 .65| 75.9 72.0| Q24 0 |

| 19 872 197 -.39 .15| .99 -.10| .82 -.83| .67 .66| 65.8 69.5| Q19 0 |

| 22 865 197 -.79 .15|1.02 .21| .83 -.85| .68 .67| 69.5 69.0| Q22 0 |

| 11 831 197 -.40 .14|1.05 .45|1.07 .53| .68 .70| 60.4 67.8| Q11 0 |

| 2 842 197 -.76 .14|1.06 .46|1.01 .13| .69 .68| 77.5 69.3| Q2 0 |

| 6 869 197 -.85 .15| .87 -1.07| .81 -1.02| .70 .66| 73.8 70.2| Q6 0 |

| 23 835 197 .07 .15| .98 -.20| .90 -.67| .72 .70| 75.4 69.2| Q23 0 |

| 12 762 197 .64 .13|1.05 .44|1.10 .89| .73 .75| 65.2 64.8| Q12 0 |

| 3 756 197 .77 .14|1.00 .01|1.04 .34| .73 .74| 63.6 68.2| Q3 0 |

| 16 699 197 .59 .13|1.14 1.21|1.17 1.55| .73 .78| 57.2 63.6| Q16 0 |

| 15 776 197 .36 .13|1.02 .19|1.01 .16| .74 .75| 61.0 63.9| Q15 0 |

| 13 812 197 -.24 .15| .88 -1.05| .87 -1.05| .74 .72| 71.7 69.5| Q13 0 |

| 21 853 197 -.16 .15| .75 -2.51| .76 -1.55| .74 .69| 75.4 69.0| Q21 0 |

| 8 801 197 .08 .14| .86 -1.20| .85 -1.28| .75 .72| 72.2 68.6| Q8 0 |

| 17 753 197 .73 .13|1.05 .53|1.05 .52| .75 .76| 66.3 62.7| Q17 0 |

| 14 819 197 .52 .14| .86 -1.41| .83 -1.33| .75 .72| 69.5 67.4| Q14 0 |

| 9 788 197 .22 .14| .86 -1.22| .87 -1.14| .76 .73| 68.4 67.9| Q9 0 |

| 4 780 197 .16 .14| .89 -.98| .90 -.87| .76 .74| 71.7 67.5| Q4 0 |

| 5 808 197 .43 .15| .79 -2.08| .77 -2.09| .78 .72| 75.9 69.4| Q5 0 |

| 10 788 197 .03 .14| .80 -1.95| .77 -2.11| .78 .74| 73.8 67.0| Q10 0 |

|------------------------------------+----------+----------+-----------+-----------+---------|

| MEAN 815.0 197.0 .00 .14|1.01 -.10|1.00 -.11| | 69.0 68.0| |

| P.SD 44.8 .0 .51 .01| .20 1.39| .27 1.51| | 5.7 2.3| |

-------------------------------------------------------------------------------------------

### Supplementary data 3: Empirical Item-Category Measures

**Student Average Measures**

Observed Average Measures for PERSON (unscored) (by Observed Category)

-7-6 -5 -4 -3 -2 -1 0 1 2 3 4 5 6 7 8 9

|--+--+--+--+--+--+--+--+--+--+--+--+--+--+--+--| NUM ITEM

| 1 2 3 4 5 | 16 Q16

| 1 2 3 4 5 | 6 Q6

| |

| 12 3 4 5 | 15 Q15

| |

| |

| 0 1 2 3 4 5 | 17 Q17

| |

| 12 3 4 5 | 13 Q13

| 1 2 3 4 5 | 12 Q12

| |

| |

| 2 3 4 5 | 10 Q10

| 2 13 4 5 | 3 Q3

| |

| 12 3 4 5 | 4 Q4

| 1 2 3 4 5 | 7 Q7

| 23 4 5 | 20 Q20

| |

| 2 3 4 5 | 8 Q8

| 1 2 3 4 5 | 9 Q9

| 2 13 4 5 | 5 Q5

| 12 3 4 5 | 11 Q11

| |

| |

| 2 3 4 5 | 14 Q14

| 2 3 4 5 | 2 Q2

| |

| |

| 32 4 5 | 21 Q21

| 2 3 4 5 | 19 Q19

| 23 4 5 | 23 Q23

| 3 4 0 5 | 1 Q1

| 34 5 | 25 Q25

| 2 3 41 5 | 24 Q24

| |

| 3 4 5 | 18 Q18

| 3 4 5 | 22 Q22

|--+--+--+--+--+--+--+--+--+--+--+--+--+--+--+--| NUM ITEM

-7-6 -5 -4 -3 -2 -1 0 1 2 3 4 5 6 7 8 9

112121122111

22368300639132821884268 6 9 PERSON

T S M S T

0 10 20 40 70 80 90 99 PERCENTILE

M = MEAN

S = 1 ST DEV

T = 2 ST DEV

**Preceptor Average Measures**

Observed Average Measures for PERSON (unscored) (by Observed Category)

-5 -4 -3 -2 -1 0 1 2 3 4 5 6 7 8

|---+---+---+---+---+---+---+---+---+---+---+---+---| NUM ITEM

| 1 2 3 4 5 | 3 Q3

| 12 3 4 5 | 17 Q17

| |

| 2 3 4 5 | 7 Q7

| 1 2 3 4 5 | 12 Q12

| 0 1 2 3 4 5 | 16 Q16

| |

| 2 3 4 5 | 14 Q14

| |

| 2 3 4 5 | 5 Q5

| |

| 1 2 3 4 5 | 15 Q15

| |

| |

| 1 2 3 4 5 | 9 Q9

| 1 2 3 4 5 | 4 Q4

| |

| 1 2 3 4 5 | 8 Q8

| 3 2 4 5 | 23 Q23

| 1 2 3 4 5 | 10 Q10

| |

| |

| 2 3 4 5 | 20 Q20

| 2 3 4 5 | 21 Q21

| 2 3 4 5 | 18 Q18

| 1 2 3 4 5 | 13 Q13

| |

| |

| 2 3 4 5 | 19 Q19

| 1 2 3 4 5 | 11 Q11

| |

| 1 2 3 4 0 5 | 1 Q1

| |

| |

| 1 3 04 5 | 2 Q2

| 2 1 3 4 5 | 22 Q22

| 2 3 4 5 | 24 Q24

| 1 23 4 5 | 6 Q6

|---+---+---+---+---+---+---+---+---+---+---+---+---| NUM ITEM

-5 -4 -3 -2 -1 0 1 2 3 4 5 6 7 8

1 11 1 1

11 313431555666892850964147273354 3 4 3 0 PERSON

T S M S T

0 10 20 30 50 60 70 80 90 99 PERCENTILE

M = MEAN

S = 1 ST DEV

T = 2 ST DEV

### Supplementary data 4: item misfit order

Students

-------------------------------------------------------------------------------------------

|ENTRY TOTAL TOTAL JMLE MODEL| INFIT | OUTFIT |PTMEASUR-AL|EXACT MATCH| |

|NUMBER SCORE COUNT MEASURE S.E. |MNSQ ZSTD|MNSQ ZSTD|CORR. EXP.| OBS% EXP%| ITEM |

|------------------------------------+----------+----------+-----------+-----------+------|

| 6 956 270 1.92 .10|1.38 3.70|1.50 4.75|A .64 .74| 47.5 57.8| Q6 |

| 7 1098 269 .39 .11|1.38 3.64|1.32 2.78|B .64 .68| 56.5 63.2| Q7 |

| 24 1222 269 -1.32 .13|1.28 2.72|1.16 .84|C .52 .57| 73.5 71.6| Q24 |

| 1 1221 270 -1.25 .13|1.07 .73|1.25 1.30|D .58 .57| 76.6 71.0| Q1 |

| 11 1142 270 -.10 .11|1.21 2.20|1.13 1.07|E .62 .65| 61.7 64.6| Q11 |

| 14 1179 270 -.60 .12|1.09 .98|1.14 1.01|F .58 .62| 65.9 67.2| Q14 |

| 17 1012 270 1.38 .10|1.14 1.42|1.09 .95|G .70 .72| 58.2 60.2| Q17 |

| 25 1223 270 -1.28 .13|1.09 .92|1.09 .50|H .54 .57| 70.9 71.3| Q25 |

| 16 945 270 2.02 .10|1.02 .26|1.07 .74|I .72 .74| 54.8 57.5| Q16 |

| 19 1216 270 -1.17 .13|1.06 .61| .88 -.60|J .58 .58| 69.0 70.6| Q19 |

| 15 979 270 1.71 .10|1.03 .38|1.05 .52|K .71 .73| 57.1 58.7| Q15 |

| 23 1217 270 -1.18 .13|1.05 .51| .99 -.01|L .54 .58| 71.6 70.7| Q23 |

| 18 1242 270 -1.61 .14|1.04 .47|1.03 .21|M .52 .55| 73.9 73.2| Q18 |

| 12 1032 270 1.18 .10| .97 -.29| .94 -.66|l .74 .71| 62.8 60.7| Q12 |

| 22 1245 270 -1.67 .14| .97 -.29| .83 -.71|k .55 .54| 76.2 73.5| Q22 |

| 8 1127 270 .09 .11| .94 -.63| .93 -.63|j .69 .66| 64.4 63.8| Q8 |

| 20 1114 270 .25 .11| .87 -1.45| .93 -.62|i .70 .67| 70.1 63.4| Q20 |

| 21 1211 270 -1.09 .13| .79 -2.42| .93 -.36|h .63 .59| 76.6 70.0| Q21 |

| 9 1138 270 -.05 .11| .89 -1.16| .91 -.73|g .69 .65| 68.6 64.3| Q9 |

| 4 1093 270 .50 .11| .87 -1.37| .84 -1.54|f .74 .68| 65.5 62.7| Q4 |

| 5 1139 270 -.06 .11| .83 -1.98| .76 -2.15|e .72 .65| 71.3 64.3| Q5 |

| 3 1077 270 .69 .11| .76 -2.74| .82 -1.83|d .74 .69| 68.6 62.1| Q3 |

| 10 1076 270 .70 .11| .82 -2.01| .79 -2.18|c .72 .69| 64.8 62.1| Q10 |

| 13 1031 270 1.19 .10| .77 -2.57| .74 -2.93|b .76 .71| 67.8 60.7| Q13 |

| 2 1183 270 -.66 .12| .71 -3.51| .63 -2.89|a .69 .62| 73.9 67.6| Q2 |

|------------------------------------+----------+----------+-----------+-----------+------|

| MEAN 1124.7 269.9 .00 .11|1.00 -.07| .99 -.13| | 66.7 65.3| |

| P.SD 90.9 .3 1.13 .01| .18 1.90| .19 1.69| | 7.4 4.9| |

-------------------------------------------------------------------------------------------

Preceptors

-------------------------------------------------------------------------------------------

|ENTRY TOTAL TOTAL JMLE MODEL| INFIT | OUTFIT |PTMEASUR-AL|EXACT MATCH| |

|NUMBER SCORE COUNT MEASURE S.E. |MNSQ ZSTD|MNSQ ZSTD|CORR. EXP.| OBS% EXP%| ITEM |

|------------------------------------+----------+----------+-----------+-----------+------|

| 1 849 197 -.52 .13|1.80 3.81|2.05 4.71|A .54 .64| 73.3 70.5| Q1 |

| 7 814 197 .66 .14|1.21 1.91|1.36 2.56|B .67 .72| 59.4 66.9| Q7 |

| 16 699 197 .59 .13|1.14 1.21|1.17 1.55|C .73 .78| 57.2 63.6| Q16 |

| 18 857 197 -.20 .14|1.14 1.32|1.10 .60|D .65 .68| 66.3 68.1| Q18 |

| 20 851 197 -.15 .15|1.14 1.30|1.14 .91|E .65 .69| 67.4 70.5| Q20 |

| 12 762 197 .64 .13|1.05 .44|1.10 .89|F .73 .75| 65.2 64.8| Q12 |

| 11 831 197 -.40 .14|1.05 .45|1.07 .53|G .68 .70| 60.4 67.8| Q11 |

| 2 842 197 -.76 .14|1.06 .46|1.01 .13|H .69 .68| 77.5 69.3| Q2 |

| 17 753 197 .73 .13|1.05 .53|1.05 .52|I .75 .76| 66.3 62.7| Q17 |

| 3 756 197 .77 .14|1.00 .01|1.04 .34|J .73 .74| 63.6 68.2| Q3 |

| 15 776 197 .36 .13|1.02 .19|1.01 .16|K .74 .75| 61.0 63.9| Q15 |

| 22 865 197 -.79 .15|1.02 .21| .83 -.85|L .68 .67| 69.5 69.0| Q22 |

| 19 872 197 -.39 .15| .99 -.10| .82 -.83|l .67 .66| 65.8 69.5| Q19 |

| 23 835 197 .07 .15| .98 -.20| .90 -.67|k .72 .70| 75.4 69.2| Q23 |

| 24 881 197 -.79 .16| .95 -.46| .84 -.78|j .67 .65| 75.9 72.0| Q24 |

| 4 780 197 .16 .14| .89 -.98| .90 -.87|i .76 .74| 71.7 67.5| Q4 |

| 13 812 197 -.24 .15| .88 -1.05| .87 -1.05|h .74 .72| 71.7 69.5| Q13 |

| 6 869 197 -.85 .15| .87 -1.07| .81 -1.02|g .70 .66| 73.8 70.2| Q6 |

| 9 788 197 .22 .14| .86 -1.22| .87 -1.14|f .76 .73| 68.4 67.9| Q9 |

| 8 801 197 .08 .14| .86 -1.20| .85 -1.28|e .75 .72| 72.2 68.6| Q8 |

| 14 819 197 .52 .14| .86 -1.41| .83 -1.33|d .75 .72| 69.5 67.4| Q14 |

| 10 788 197 .03 .14| .80 -1.95| .77 -2.11|c .78 .74| 73.8 67.0| Q10 |

| 5 808 197 .43 .15| .79 -2.08| .77 -2.09|b .78 .72| 75.9 69.4| Q5 |

| 21 853 197 -.16 .15| .75 -2.51| .76 -1.55|a .74 .69| 75.4 69.0| Q21 |

|------------------------------------+----------+----------+-----------+-----------+------|

| MEAN 815.0 197.0 .00 .14|1.01 -.10|1.00 -.11| | 69.0 68.0| |

| P.SD 44.8 .0 .51 .01| .20 1.39| .27 1.51| | 5.7 2.3| |

-------------------------------------------------------------------------------------------
